# Bond strength of different viscosities Giomer-based composites to dentin under erosive conditions

**DOI:** 10.3389/fdmed.2026.1844321

**Published:** 2026-07-21

**Authors:** Maria Laura Diniz Pereira, Kusai Baroudi, Apurva Mishra, José Alejandro Castro González, Bruno Silva Neto, Gabriel Florio Cairo, Alessandra Bühler Borges, Rayssa Ferreira Zanatta

**Affiliations:** 1 Department of Restorative Dentistry, School of Dentistry, University of Taubaté (UNITAU), Taubaté, Brazil; 2 Department of Clinical Sciences, College of Dentistry, Ajman University, Ajman, United Arab Emirates; 3 Department of Dentistry, University of Brasília (UnB), Brasília, DF, Brazil; 4 School of Dentistry, Paulista Association of Dental Surgeons (APCD/FAOA), São Paulo, Brazil; 5 Department of Restorative Dentistry, Institute of Science and Technology, Sao Paulo State University, Sao Jose dos Campos, SP, Brazil; 6 Department of Dentistry and Dental Materials, Federal University of Uberlandia, Uberlandia, Brazil

**Keywords:** composite resin, dental bonding, dental materials, dentin, tooth erosion

## Abstract

**Objectives:**

The present study aimed to evaluate the bond strength of Giomer-based restorative composites with different viscosities bonded to dentin under various erosive conditions.

**Materials and methods:**

One hundred and twenty dentin specimens from human sound molars were selected and divided into three groups according to the erosive challenge: ERO (initial erosion with citric acid 0.3%, pH 2.6, 5 min, 5x/day, 1 day), CYC (ERO protocol, followed by an additional 5-day erosive cycling challenge after restoration), and CON (sound dentin, without erosion, control). Specimens were further subdivided into four groups (*n* = 10) according to the Giomer-based restorative material used: flowable composite (FC), flowable bulk-fill composite (FBF), regular-viscosity composite (CC), and regular-viscosity bulk-fill composite (CBF). Two cylinders (1.5 mm diameter) with the resins from each group were made in each sample, after applying the two-step self-etching adhesive system. In the CYC group, the erosive cycle was carried out before and after placement of the adhesive system and composite cylinders. Bond strength was assessed by the micro shear test, and failure was quantified in a stereomicroscope. The data were submitted to two-way ANOVA, followed by the Tukey test (*p* < 0.05).

**Results:**

Significant differences were observed for the eroded substrate (*p* = 0.010) and the composite resins (*p* = 0.037), but not for their interaction (*p* = 0.677). The CYC groups showed lower bond strength values than control group, and only the FC composite presented lower values than FBF, while CC and CBF showed similar values.

**Conclusion:**

Erosive cycling decreased the bond strength for all composites tested, irrespective of their viscosity. Regarding the flowable composites, the bulk fill one presented a higher value than the conventional non-bulk fill, while this difference was not observed for the regular-viscosity composites.

## 1 Introduction

Erosive tooth wear (ETW) is a multifactorial condition influenced by biological, behavioral, and environmental factors, and is characterized by chemical and physical loss of dental hard tissues in the absence of biofilm (1). The condition has gained increasing attention in recent years, as its incidence and prevalence have risen significantly, particularly among younger individuals (2, 3). ETW lesions first appear as shallow defects with a dull surface, which may progress to more advanced structural loss, leading to dentin exposure and even symptoms such as dentin hypersensitivity (4, 5), with potential negative impacts on the quality of life (6). Depending on the severity of tissue loss, restorative procedures may become necessary to reestablish form, function, and esthetics. In such cases, adhesive restorative approaches are preferred (4, 7).

The success of restorative interventions in managing ETW depends not only on the integrity of the adhesive interface between restorative materials and dental substrates, but also on the extent of tooth surface loss and on the physical and mechanical properties of the selected material, such as resistance to wear and chemical degradation (7, 8). While restorations can reestablish function and esthetics and may help reduce the rate of progression, they should not be considered a definitive treatment for ETW. Management should primarily aim at identifying and controlling the etiological factors driving the condition, with restorative procedures serving as adjunctive measures to restore compromised structures and maintain oral health.

Resin composites, conventional glass ionomer cements (GICs), and their hybrids are the restorative materials most frequently employed for ETW lesions (9, 10). GICs are often used due to their ability to chemically bond to tooth structures, their coefficient of thermal expansion similar to dentin, and their fluoride release, which may reduce demineralization of adjacent enamel and dentin (4, 11, 12). Nevertheless, GICs show lower resistance to erosive and abrasive challenges, resulting in higher surface wear and reduced longevity compared with resin composites (4, 12, 13). Resin composites, on the other hand, exhibit superior resistance to acid degradation (10, 14). To combine this durability with bioactive functionality, a proprietary technology employing surface pre-reacted glass (S-PRG) fillers was developed. These fillers, produced from fluoroboroaluminosilicate glass treated with polysiloxane and subsequently exposed to polyacrylic acid, are capable of releasing multiple ions, including fluoride, silicate, aluminum, sodium, borate, and strontium and are incorporated into restorative materials known as Giomers (15–17).

Like conventional resin composites, Giomer-based restoratives are available in multiple formulations for different clinical applications, including regular and bulk-fill versions, each with conventional or low-viscosity (flowable) consistencies. The incorporation of S-PRG fillers aims to provide additional benefits not usually associated with resin-based materials, such as inhibition of microbial growth, reduction of bacterial adhesion, buffering of local pH fluctuations, and inhibition of mineral loss in adjacent hard tissues, potentially reducing the risk of secondary caries (16, 18–20). These features make Giomer technology an attractive restorative alternative for patients at elevated risk of ETW, as it may offer enhanced protection for surrounding tissues during erosive episodes. However, although some evidence indicates that Giomers can withstand acidic conditions similarly to conventional composites, their protective potential in preventing additional tissue loss under frequent erosive challenges remains inconclusive (4, 21).

Despite advancements in restorative resin technology, limitations such as polymerization shrinkage, marginal leakage, and interfacial stress remain concerns, particularly in posterior restorations (22). The introduction of bulk-fill composites, with modified monomer and initiator systems, aimed to reduce shrinkage stress while allowing deeper increments and shorter application times (23). Both bulk-fill and conventional composites are also offered in flowable formulations, designed to improve margin adaptation and handling (24, 25). Conventional high-viscosity resin composites are traditionally placed incrementally to ensure adequate depth of cure and to limit polymerization-related stress. Bulk-fill composites were developed to permit placement in thicker increments and reduce clinical application time through modifications in composition and curing behavior (24). Flowable formulations exhibit lower viscosity and, in many cases, lower filler loading, which may favor adaptation to cavity walls and reduce void formation, although these same features may influence their mechanical behavior and polymerization shrinkage (24, 26). Accordingly, differences in filler loading, resin matrix composition, viscosity, and curing behavior among conventional, bulk-fill, and flowable restorative materials may affect both mechanical and adhesive performance (24–26). Giomer-based restorative materials are likewise available in these configurations, but variations in filler load and viscosity may affect their adhesive performance, particularly under erosive conditions. Given that long-term success in ETW cases relies on stable adhesion to eroded dentin, evaluating the bond strength of Giomer composites with different viscosities under erosive challenges is important. Therefore, this study aimed to investigate if different viscosities of Giomer restorative composites interfere with its bond strength to dentin under erosive cycling. The null hypothesis tested was that no significant differences would be observed in bond strength among the tested Giomers under erosive conditions.

## 2 Materials and methods

### 2.1 Ethical procedures and experimental design

This study was approved by the local University Ethics Committee (protocol number 3.236.759/CAAE: 10242919.8.0000.8447). Human sound molars extracted for therapeutic reasons in the university clinics were collected and subsequently donated for research after patients provided informed consent. All procedures were conducted in accordance with the ethical principles outlined in the Declaration of Helsinki (World Medical Association). The specimens were prepared and randomly allocated into twelve experimental groups (*n* = 10 per group) according to two factors: (1) the Giomer composite resin formulation tested—regular viscosity, regular bulk-fill, flowable, and flowable bulk-fill; and (2) the substrate condition—sound dentin (control), previously eroded dentin (ERO), and eroded dentin subjected to additional erosive cycling (CYC). The sample size was determined based on a pilot study and calculated using a standard sample size calculation tool provided by the Statistical Department of the University of São Paulo (USP-Bauru, Brazil). The calculation indicated a minimum of 8 specimens per group. To compensate for possible specimen loss and increase the reliability of the results, 10 specimens were included in each experimental group.

### 2.2 Specimen preparation

**Figure 1:**
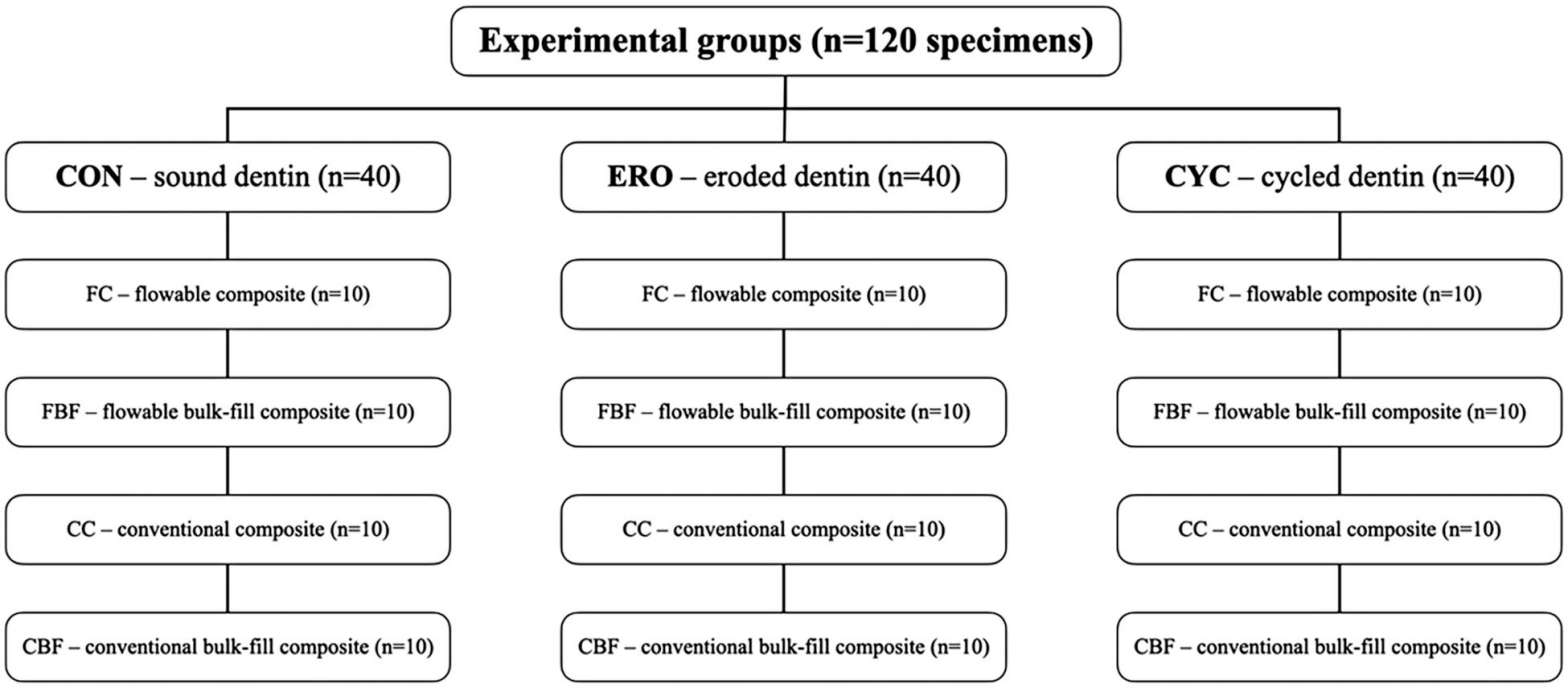
Experimental groups were divided by material—FC, FBF, CC, and CBF—and by substrate condition: CON (sound dentin), ERO (eroded dentin), and CYC (cycled) (Each subgroup had *n* = 10).

One hundred and twenty sound molars were selected, cleaned, and kept in a 0.1% thymol solution until use. They had their occlusal surface cut in the mesial-distal direction to expose the dentin. Then, teeth were embedded in epoxy resin using PVC tube (25 mm diameter; Tubos Tigre, Brazil), and ground flat using aluminum oxide abrasive paper (grit #600—Extec Corp, CT, USA) in a polishing machine (Aropol E, Arotec, São Paulo, Brazil) under running water irrigation for 30 s. Teeth were randomly divided into 3 groups (*n* = 40) according to the erosive condition: CON—sound dentin (no erosion), ERO—dentin subjected to initial erosion; and CYC-dentin subjected to initial erosion, followed by another erosive cycling after restoration (Figure 1). Specimens in ERO and CYC groups underwent an initial erosive protocol using a controlled pH-cycling model based on repeated citric-acid challenges. Specimens were immersed in 0.3% citric acid (pH 2.6, at room temperature) for 5 min, 5 times. Between erosive episodes, specimens were immersed in artificial saliva for 60 min (37 °C), between each acid challenge to allow a remineralization phase. Solutions were renewed at each immersion, and specimens were stored in fresh artificial saliva overnight. Specimens in the CON group were stored in deionized water at 37 °C for 24 h before testing.

The specimens were then randomly subdivided into four groups (*n* = 10), according to resin composite tested: FC—flowable composite (Beautifil II flow, Shofu); FBF—flowable bulk-fill composite (Beautifil Bulk Flow, Shofu, Japan); CC—conventional composite (Beautifil II, A2 shade, Shofu, Japan), and CBF—conventional bulk-fill composite (Beautifil Bulk Restorative, A2 shade, Shofu, Japan). All composite resins tested presented the Giomer technology, with S-PRG fillers, and their composition is described in Table 1. Figure 1 shows the schematic division of the groups. The study compared only the bond strength of S-PRG restorative composites with different viscosities under erosive conditions. It excluded restorative materials without S-PRG technology and did not evaluate the isolated effect of S-PRG fillers.

**Table 1:** Composition of the materials tested.

Material	Group	Composition
Beautifil flow plus F00 (Shofu-Japan)	*FC*-flowable composite	Monomers: Bis-GMA, TEGDMA
		Filler: S-PRG filler (67.3 wt%). Filler size: 0.01–4 μm. Mean filler size: 0.8 μm
Beautifil Bulk Flow (Shofu-Japan)	*FBF-*flowable bulk-fill composite	Monomers: Bis-GMA, UDMA, Bis-MPEPP, TEGDMA.
		Filler: S-PRG filler (73 wt%). Filler size: not reported by manufacturer
Beautifil II (Shofu-Japan)	*CC-*conventional composite	Monomers: Bis-GMA, TEGDMA.
		Filler: Total filler content 83 wt% (including S-PRG filler). Filler size: 0.01–4 μm; mean filler size: 0.8 μm.
Beautifil Bulk Restorative (Shofu-Japan)	*CBF-*conventional bulk-fill composite	Monomers: Bis-GMA, UDMA, Bis-MPEPP, TEGDMA.
		Filler: S-PRG filler (87 wt%). Filler size: not reported by manufacturer.
FL-BOND II—Adhesive System (Shofu-Japan)		Primer: Carboxylic acid monomer (4-META), Phosphonic acid monomer (6-MHPA), Water, Ethanol (solvent), Photo-initiator.
		Adhesive: HEMA, UDMA, TEGDMA, multifunctional glass filler (40% wt, S-PRG filler), Photo-initiator.

Bis-GMA, bisphenol A-glycidyl methacrylate; UDMA, urethane dimethacrylate; Bis-MPEPP, 2,2-bis[(4-methacryloxypolyethoxy)phenyl] propane; TEGDMA, triethylene glycol dimethacrylate; S-PRG, surface pre-reacted glass-ionomer (fluoroboroaluminosilicate); 2-HEMA, 2-hydroxyethyl methacrylate; 6-MHPA, 6-methacryloxyhexyl 3-phosphonoacetate; 4-META, 4-methacryloxyethyl trimellitate anhydride; Photo-initiator, polymerization initiator; solvent, organic vehicle; water, aqueous component of primer.

The dentin substrate was treated only with a two-step self-etching adhesive (FL-BOND II—Shofu, Japan), which also contains S-PRG fillers as described in Table 1. No additional surface pretreatment was performed on eroded dentin before adhesive application. This approach was adopted to standardize the bonding protocol across groups and isolate the effect of erosive condition and Giomer composite viscosity on bond strength. The primer was actively applied for 10 s, followed by a gentle air blast for 5 s, and was then distanced 10 mm from the sample to evaporate solvent. Then, the bond was also actively applied, followed by a gentle air blast and then light cured with an LED light (Bluephase, Ivoclar Vivadent, Schaan, Liechtenstein, Standard irradiance, 1,400 mW/cm^2^), for 10 s. After that, the material of each group was placed with the help of a plastic cylinder (1.5 mm in diameter and 2 mm in height) in a single increment and light-cured for 20 s. Each specimen received two cylinders of the same material, with a minimum distance of 1 mm between them. Furthermore, care was taken to prevent material from leaking out of the tube to avoid interfering with the adhesive strength. Then, the specimens were stored for 7 days under controlled temperature (37 °C) in containers with a small amount of water at the bottom, used only to maintain humidity, without covering the samples completely.

### 2.3 Erosive cycling

After 7 days of cylinders placement, the specimens from the CYC group were submitted to an additional 5-day erosive cycling protocol. Erosion was induced using a controlled pH-cycling model based on repeated citric-acid challenges. Specimens were immersed in 0.3% citric acid (pH 2.6) for 5 min, 5 times/day. Between erosive episodes, specimens were immersed in artificial saliva [pH 7.0; CaCl_2_·2H_2_O 0.1029 g/L, MgCl_2_ 0.04066 g/L, KH_2_PO_4_ 0.544 g/L, KCl 2.2356 g/L, tris(hydroxymethyl)-aminomethane 4.766 g/L, and distilled water up to 1,000 mL] (40) for 60 min (5 times/day) to allow a remineralization phase. Solutions were renewed at each immersion, and specimens were stored in fresh artificial saliva at 37 °C overnight. The specimens of the CON and ERO groups remained stored in artificial saliva at 37 °C during the same period.

### 2.4 Bond strength test

The bond strength was measured by a microshear test, using a universal testing machine (Mbio I, BioPDI, São Carlos, Brazil) with 1 mm/min speed. The mean value between the two cylinders was considered for statistical analysis. The bond strength (BS) was calculated in MPa using the load at failure F (N), and the adhesive area A (mm^2^), using the formula BS = F/A. For the definition of the bonding area, the cylinder diameter was checked with the help of a digital caliper, and the area was calculated using the formula *A* = *π*.*r*^2^. The value of *π* was defined as 3.14.

After shear test, failure patterns were evaluated under 20× magnification and classified into four categories: adhesive failure (failure at the adhesive interface), cohesive failure in resin (failure within the resin composite), cohesive failure in dentin (failure within dentin), and mixed failure (failure involving both dentin and resin).

### 2.5 Statistical analysis

The dentin specimen was considered the experimental unit. For each specimen, micro-shear bond strength was calculated as the mean of the two cylinders. When pretest failure (PTF) occurred, the failed cylinder was assigned as 0 MPa, and the specimen mean was calculated using the tested cylinder value and the 0 MPa value to avoid overestimation associated with excluding prematurely failed specimens. Homogeneity of variances and normal distribution of data were checked using the Levene and Shapiro–Wilk tests, respectively. Considering that both criteria were met, the parametric analysis of variance in two factors was applied—Two-way ANOVA (factors composite resin and erosive protocol), followed by Tukey's *post hoc* test, considering *p* < 0.05. All tests were performed with Jamovi software (version 2.0; The Jamovi Project, Sydney, Australia, 2021). The results from failure analysis were evaluated qualitatively.

## 3 Results

The results from the two-way ANOVA showed significant differences for the eroded substrate (*p* = 0.010) and the composite resins (*p* = 0.037), but not for their interaction (*p* = 0.677). Table 2 shows the bond strength mean values and standard deviation for all composites and aging protocols tested, and results from Tukey's test. Specimens subjected to additional erosive cycling (CYC) showed overall significantly lower bond strength than the control group (CON). The flowable bulk-fill composite (FBF) showed the highest overall bond strength values, whereas the conventional flowable composite (FC) showed the lowest values. The conventional composite (CC) and conventional bulk-fill composite (CBF) exhibited intermediate performance and were not statistically different from either FBF or FC.

**Table 2:** Bond strength means and standard deviation (MPa) for all composites and erosive protocol tested, and results from Tukey's test.

Composite resin	ERO		CYC		CON		Composite resin factor
	Mean	SD	Mean	SD	Mean	SD	
FC	12.8	(±2.7)	9.4	(±6.6)	16.7	(±5.0)	B
FBF	17.8	(±6.4)	16.8	(±5.6)	19.1	(±5.6)	A
CC	17.4	(±5.7)	12.1	(±7.5)	14.6	(±5.3)	AB
CBF	17.2	(±6.7)	13.1	(±6.4)	19.6	(±8.5)	AB
Substrate factor	ab		b		a		

Uppercase letters show differences within the composite resin factor in the column. Lowercase letters show differences within the eroded substrate in the line.

**Figure 2:**
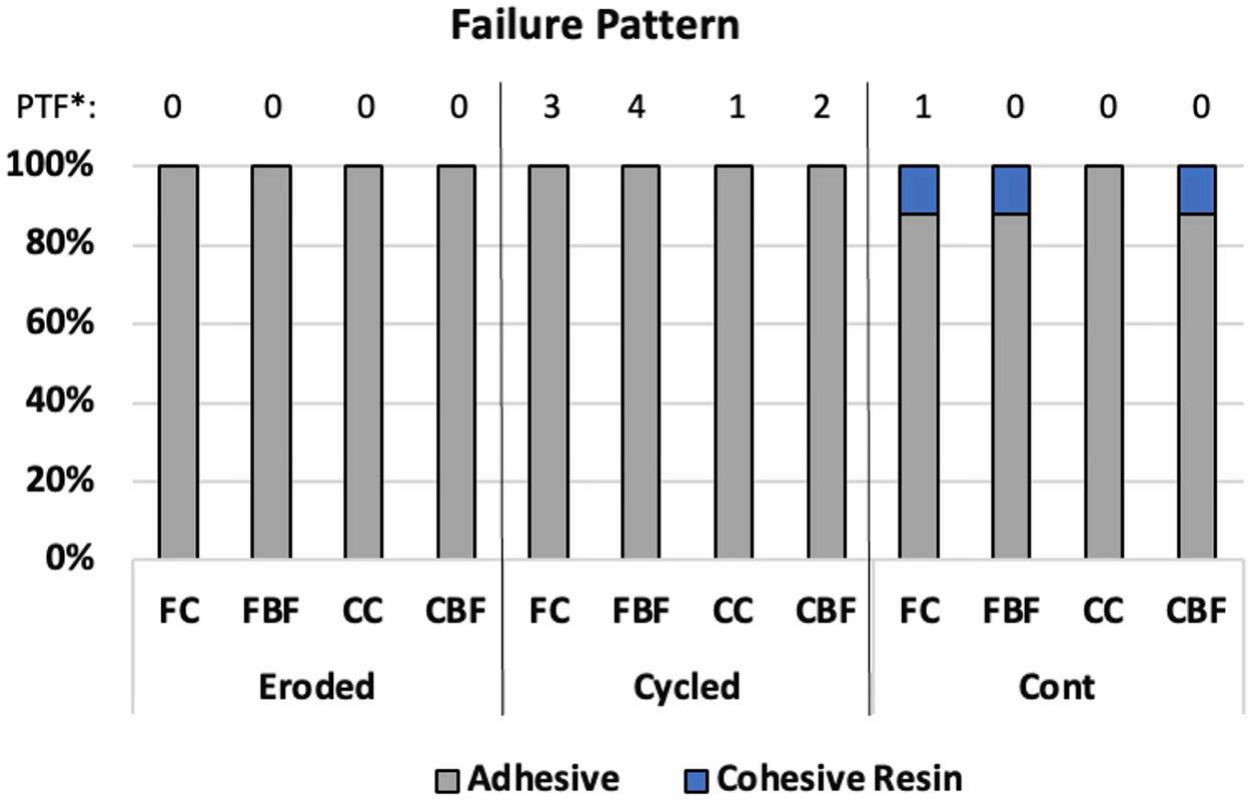
Failure pattern distribution among groups. Failure patterns were classified as adhesive failure and cohesive failure in resin. PTF, pre-test failures.

Regarding failure patterns, most groups presented predominantly adhesive failures (Figure 2), and only the groups FC, FBF, and CBF from the sound condition (control groups) presented a cohesive failure in the composite. Interestingly, it shall be noticed that the groups from the CYC group presented several specimens in which one of the cylinders had pre-test failure (PTF) and, therefore, their values were considered null for statistical purposes. In the cycled group, the conventional composite presented three pre-test failures, the conventional flow presented four, the bulk flow presented one, and the bulk fill presented two, as shown in Figure 2. From the control group, only the CC group presented a single pre-test failure (PTF) (Figure 2). For all other groups, no pre-test failures occurred. No specimens presented PTF in both cylinders.

## 4 Discussion

The influence of erosive conditions over the adhesive interface has been pointed out as an important factor to determine the success of the restorative procedure, with diverging results in the literature (8, 27, 28). This research evaluated the dentin bond strength of four composites containing the S-PRG fillers (Giomer technology) and different viscosities, under erosive conditions. Despite no differences being found in their interaction, erosive cycling decreased bonding compared to sound dentin. Overall, the flowable composite (FC) showed lower dentin bond strength values than the flowable bulk fill composite (FBF); therefore, the null hypothesis was rejected.

Erosion is known to modify dentin substrate characteristics and impair adhesive bonding, and systematic evidence indicates that surface pretreatments targeting the demineralized collagen layer may improve bond strength to eroded dentin (29). In the present experimental design, no pretreatment was performed in order to preserve a standardized bonding protocol and isolate the comparison among Giomer restorative systems with different viscosities. Therefore, the bond strength results should be interpreted as the performance of these restorative systems under a direct bonding approach over eroded dentin rather than as evidence of a specific topography-mediated mechanism, and differences among erosive conditions were inferred based on the standardized pH-cycling protocol rather than directly validated by surface measurements. A limitation of the present study is the absence of complementary structural and microstructural characterization of the materials tested and treated dentin surfaces. Although the bonding procedures were standardized, analyses such as scanning electron microscopy, surface roughness and profile (measured through profilometry), or chemical/morphological characterization could have provided additional insight into the relationship between surface morphology, material structure, and bond strength.

Recent clinical trials evaluating composites with the S-PRG fillers showed promising results in terms of retention rates (30, 31), and a recent study showed adequate bonding ability to carious affected dentin (32) and demineralized enamel (33), but evidence under erosive conditions is still scarce. The promising performance of the Giomer composites on carious dentin is attributed to their ability to form a more regular reinforcement of the demineralized dentin by the fluoride and silicate ions, improving its surface energy and wettability (32, 34). In the present study, the similar bond strength observed between sound and initially eroded dentin may be related to the characteristics of the erosion protocol. The initial erosive challenge may not have been severe enough to impair the interaction between dentin and the adhesive system, especially considering that the same adhesive protocol was used for all groups. In contrast, the additional erosive cycling performed after restoration negatively affected bond strength, suggesting that repeated acid exposure may compromise the bonded interface over time, regardless of composite viscosity. Differences among composites may be associated with their specific formulations, including possible variations in the amount and distribution of S-PRG fillers. Because ion release was not directly measured in the present study, this interpretation remains speculative. Future studies should include ion-release analysis and complementary surface or microstructural characterization to better clarify the role of material composition in bonding to eroded dentin.

The higher frequency of pre-test failures (PTFs) observed in the cycled groups may indicate greater interfacial vulnerability following repeated erosive challenges. Because excluding PTFs may artificially increase mean bond strength by removing the weakest interfaces, these failures were conservatively assigned a value of 0 MPa and retained in the statistical analysis. This interpretation is further supported by the greater occurrence of PTFs in the FC group, which also exhibited the lowest bond strength values. The FC composite had the lowest filler content among the tested materials (Table 1), which may have affected its performance under erosive challenges. However, other factors like monomer composition, viscosity, and filler characteristics likely influenced the results.

Additionally, the FC composite was the only one presenting a significant difference in comparison to the flowable bulk-fill (FBF), when considering only the composite type. The conventional composite (CC) and conventional bulk-fill composite (CBF) exhibited intermediate bond strength values and were not statistically different from either FC or FBF. Besides the lower number of particles, the bulk-fill one presented monomers responsible for reducing its viscosity and shrinkage, such as Bis—MPEPP and UDMA (35). It is advocated that using a flowable composite as a liner should result in better marginal adaptation and decrease of microleakage and voids, leading to a more stable restoration (26), our results indicate that for these cases a flowable bulk-fill with Bis–MPEPP could be an option for individuals with high risk for erosion.

The use of the same adhesive system in all groups was intended to standardize the bonding protocol and allow comparison among the restorative composites and erosive conditions. Nevertheless, because the adhesive also contains S-PRG fillers, its use may have reduced differences among restorative materials and limits the extrapolation of these findings to adhesive systems with different compositions. Therefore, the present results should be interpreted within the scope of bond strength performance only. Furthermore, the absence of restorative materials and adhesive systems without S-PRG fillers as comparison controls is an important limitation, and the present findings should be interpreted as the performance of the tested Giomer restorative systems rather than as evidence of a specific S-PRG-mediated effect. Future studies, including non-S-PRG restorative materials and adhesive systems, are necessary to better isolate the contribution of S-PRG technology to adhesive performance under erosive conditions.

Still, this study used a standardized citric-acid pH-cycling model to generate reproducible erosive dentin conditions and enable controlled comparisons among Giomer composites with different viscosities under identical adhesive procedures. Such models are commonly adopted in laboratory research because they provide consistent demineralization challenges interspersed with remineralization phases (36–38). However, the clinical expression of ETW is multifactorial and influenced by variables not evaluated in this study, including acquired pellicle, dietary acid diversity, salivary clearance dynamics, abrasion, thermal changes, and enzymatic/proteolytic activity that may accelerate degradation of the demineralized collagen matrix and the hybrid layer (39). Therefore, extrapolation to clinical performance should be made with caution, and future studies should incorporate combined erosion–abrasion protocols and biologically enriched conditions to improve translational relevance. Finally, it should be highlighted that proteolytic enzymes (e.g., pepsin) play an important role in the degradation of the organic matrix of eroded dentin (8), although it was not evaluated in this study. Still, further analysis should include more severe aging protocols to check susceptibility to the hybrid layer water degradation, and also adhesives with different compositions and bonding strategies (self-etch and etch-and-rinse; with and without S-PRG fillers). The aging strategy adopted herein represents a short-term erosive challenge and should not be interpreted as a proxy for long-term clinical performance. Despite that, our results are promising in showing that the bulk fill composite might be viable for restoring ETW lesions with dentin involvement and might guide future clinical trials.

## 5 Conclusion

Within the limitations of this study, it might be concluded that Giomer composites tested exhibited a reduction in bond strength to dentin after erosive cycling. Regarding viscosity, the flowable bulk fill one presented higher bond strength values than the flowable one, while this difference was not observed for the regular-viscosity composites. These findings are primary and suggest that under erosive situations regular composites might work better than flowable ones and should be confirmed on clinical trials. Also, confirmation under long-term and other aging conditions (friction and fatigue) and in clinical studies is still needed.

## Data Availability

The raw data supporting the conclusions of this article will be made available by the authors, without undue reservation.
